# Prognosis and treatment of non-metastatic primary and secondary breast angiosarcoma: a comparative study

**DOI:** 10.1186/s12885-017-3292-7

**Published:** 2017-04-27

**Authors:** Ming Yin, Wenge Wang, Joseph J. Drabick, Harvey A. Harold

**Affiliations:** Division of Hematology and Oncology, Penn State Hershey Cancer Institute, Hershey, USA

**Keywords:** Breast sarcoma, Clinicopathological characteristics, Treatment, Prognosis

## Abstract

**Background:**

Breast angiosarcoma is a rare malignancy with limited publications confined to small retrospective case reviews and case reports. Knowledge of this disease is limited because information from previous studies is insufficient and inconsistent.

**Methods:**

We obtained data from the Surveillance, Epidemiology, and End Results Program for non-metastatic primary and secondary breast angiosarcoma, and performed analysis to determine clinicopathological characteristics and estimate their associations with overall survival (OS).

**Results:**

Median age was 50–54 years in primary breast angiosarcoma and 70–74 years in secondary breast angiosarcoma, while median OS was 93 and 32 months, respectively. Age, tumor grade and tumor spread were associated with poor survival outcomes. Compared with primary breast angiosarcoma, patients with secondary breast angiosarcoma had a “nominal” increased death risk (HR = 1.89, 95% CI, 1.43–2.50, *p* < 0.001), which was driven by older age and more aggressive tumor phenotype at presentation. Mastectomy was associated with worse OS compared with breast conservative surgery (BCS) (adjHR = 2.47, 95% CI, 1.29–4.74) in primary angiosarcoma patients. Adjuvant radiation was associated with worse OS in secondary angiosarcoma patients (adjHR =1.77, 95% CI, 1.01–3.12).

**Conclusions:**

There is a “nominal” increased death risk in secondary breast angiosarcoma due to advanced clinicopathological features. Both BCS and mastectomy are feasible in primary and secondary angiosarcoma if R0 can be achieved. Routine radiation in unselected breast angiosarcoma should be cautious because there is no survival benefit in primary AS and appeared to be associated with a worse OS in secondary AS.

**Electronic supplementary material:**

The online version of this article (doi:10.1186/s12885-017-3292-7) contains supplementary material, which is available to authorized users.

## Background

Breast sarcoma, excluding phyllodes tumor, is an extremely rare and heterogenous group of malignancies, accounting for less than 1% of total breast malignancies and less than 5% of all soft tissue sarcomas [1]. The annual incidence of breast sarcomas was 4.48 cases per million women. Of all the breast sarcomas, angiosarcoma (AS) is the main histology type and carries a poor prognosis. It is a very aggressive malignant tumor of the vascular endothelium, characterized by rapidly proliferating and extensively infiltrating growth. Based on its etiology, it can be divided into two categories: de novo development (primary) or therapy-related development (secondary).

Given the rarity of the disease, the majority of studies have focused on clinicopathological features of primary breast AS, while studies of secondary breast AS are extremely limited. Excluding review articles, a literature search of Pubmed database yielded 79 primary breast AS and 26 secondary breast AS articles, most of which were small retrospective case reviews and case reports. Optimal care of breast AS is poorly defined because information from previous studies is insufficient and inconsistent. Basically, surgery is the mainstay of treatment for curative intent. Both radiotherapy and chemotherapy have been used, but treatment indications have not been clearly defined. There is concern that radiation treatment in previous irradiated area or around vulnerable tissues from previous surgery may produce serious side effects or treatment-related complications, such as rib fracture, pneumonitis, soft tissue necrosis, and delayed wound healing [2]. Indeed, current treatment recommendations are derived from small retrospective studies of breast sarcomas and do not differentiate secondary breast AS from primary AS. Although secondary AS shares many similarities with primary AS, there is evidence that the etiology and natural history of secondary breast AS may differ significantly from primary AS. For example, primary breast AS typically occurs in younger women without previous history of mammary carcinoma, while secondary breast AS is usually found in older women as a complication of radiotherapy (RT) or chronic lymphedema after lumpectomy or mastectomy. Primary AS is a malignant vascular neoplasm that arises within the breast parenchyma, while secondary AS usually arise in the cutaneous tissue and might invade the breast parenchyma secondarily. In most series, patients with secondary AS had significantly shorter survival time than those who had primary AS, and were more prone to local and distant recurrence.

The current study utilizes a population database supported by the National Cancer Institute of the US to analyze a large series of women diagnosed of primary and secondary AS. The primary objective of this study was to determine clinicopathological characteristics of the patient population with breast AS and identify patient, pathologic, and treatment characteristics that predict survival outcomes.

## Methods

### SEER database

Data for analyses were obtained from the Surveillance, Epidemiology, and End Results Program (SEER) using the dataset of SEER 18. Patients diagnosed with breast angiosarcoma between 1973 and 2012 were identified by ICD-O-3 codes. Variables included patient age at diagnosis, race, survival status, and year of diagnosis. Treatment data included extent of surgery and use of radiotherapy. The historic SEER stage was used to determine local, regional and distant tumor stage. Pathologic characteristics included tumor anatomic site, and tumor grade. Although grading of angiosarcoma is not recommended by American Joint Committee on Cancer (AJCC) 7 soft tissue sarcoma staging system, the SEER database does report tumor differentiation by well-, moderate-, poorly-, un-differentiated and unknown differentiation. We grouped poorly- and un-differentiated together to fit the 3-tier grading system used by AJCC 7. Of note, the SEER database reports age by groups with 5-year interval (e.g. 60–64 years). Therefore, age in this study was reported in a similar manner.

### Study population

We included patients with non-metastatic primary breast AS, defined as women with only one primary breast angiosarcoma and women with breast angiosarcoma as the first identified malignancy if there were multiple primary cancers in their lifetime.

We included women with breast angiosarcoma who had prior history of breast cancer, lung cancer, Hodgkin lymphoma and soft tissue cancer in the chest for analysis of secondary breast AS. Those patients likely developed breast AS as a result of prior radiation or surgery in the chest. We excluded patients with other cancer types prior to breast angiosarcoma, such as skin cancer, gastrointestinal cancer, genitourinary cancer, brain tumor, bone cancer, and gynecological cancer, because it is difficult to correlate breast angiosarcoma with the treatment they received in the past. If more than one breast angiosarcoma were documented for the same patient in SEER database, we selected the first breast angiosarcoma in our study.

### Statistical analysis

Clinical outcomes of overall survival (OS) as reported in SEER database were used for assessment. Chi-square test was performed to test the difference of distribution between groups. Cox proportional hazards regression analysis was performed to calculate the hazard ratio (HR) and 95% confidence interval (CI) to evaluate the influence of patient, pathological and treatment-related characteristics on death risk. Multivariate Cox regression was performed to adjust for other covariates. Kaplan-Meier curve was used to visualize the cumulative survival probability. Statistical analysis was performed using SAS 9.1 statistical software (SAS Inc., Chicago, IL). A *p* value of 0.05 or less was considered statistically significant.

## Results

The SEER database consists of 472 primary or secondary non-metastatic angiosarcoma patients. Angiosarcoma constitutes about 31.7% of all primary breast sarcoma and about 70.9% of secondary breast sarcoma without metastasis. Those numbers were extrapolated by the percentage of angiosarcoma in breast sarcoma without or with prior cancer history, because it is difficult to determine the exact number of therapy-related breast sarcoma if patients ever received surgery or radiation. After applying the strict inclusion criteria as described in Methods, we identified a total of 218 non-metastatic primary breast AS patients and a total of 173 non-metastatic secondary breast AS patients for further analyses.

### Comparison of patient characteristics of primary and secondary breast AS

As shown in Table 1, there was significantly different distribution between primary and secondary breast AS in each parameter group. Compared with primary AS patients, patients with secondary AS were 20 years older at diagnosis with a median age of 70–74 years. There were substantially higher number of patients with overlap or entire involvement of the breast in secondary AS (121/173, 69.5% versus. 127/218, 58.3%). Examinations of tumor stage and tumor grade also showed more locally advanced stage and high grade of tumors in patients with secondary breast AS, compared with patients with primary breast AS (regional stage: 57.8% versus. 17.9%; grade 3: 57.8% versus. 32.1%). These results suggested that secondary breast AS had more aggressive tumor phenotype than primary AS at presentation.

**Table 1:** Comparison of patient characteristics

Parameters	Primary (%)	Secondary (%)	*p*
Age (years)			
Median	50–54	70–74	
< 60	141 (64.7)	29 (16.8)	
≥ 60	77 (35.3)	144 (83.2)	<0.001
Race			
Non-white	40 (18.3)	13 (7.5)	
White	178 (81.7)	160 (92.5)	0.002
Site			
Outer quandrant	40 (18.3)	21 (12.1)	
Inner Quandrant	37 (16.9)	15 (8.7)	
Central	14 (6.4)	12 (6.9)	
Overlap^a^	62 (28.4)	31 (17.9)	
NOS^b^	65 (30)	90 (52)	
Nipple	0 (0)	4 (2.4)	<0.001
Tumor spread^c^			
Local	179 (82.1)	84 (48.6)	
Regional	39 (17.9)	89 (51.4)	<0.001
Tumor grade			
G1	34 (15.6)	13 (7.5)	
G2	54 (24.8)	22 (12.7)	
G3	70 (32.1)	100 (57.8)	
Gx	60 (27.5)	38 (22)	<0.001
Number of primaries^d^			
1	179 (82.1)	0 (0)	<0.001
2	33 (15.1)	111 (64.2)	
≥ 3	6 (2.8)	62 (35.8)	

^a^More than one quandrant but less than half breast size

^b^Entire breast: ¾ or more of breast involved with tumor, or multiple tumors in different subsites

^c^Local: tumor spread limited to the organ of origin; regional: beyond the limit of the organ of origin

^d^Number of different primary tumors developed in an individual by the time of last data collection in SEER database

### Patient characteristics and survival outcomes

**Fig. 1:**
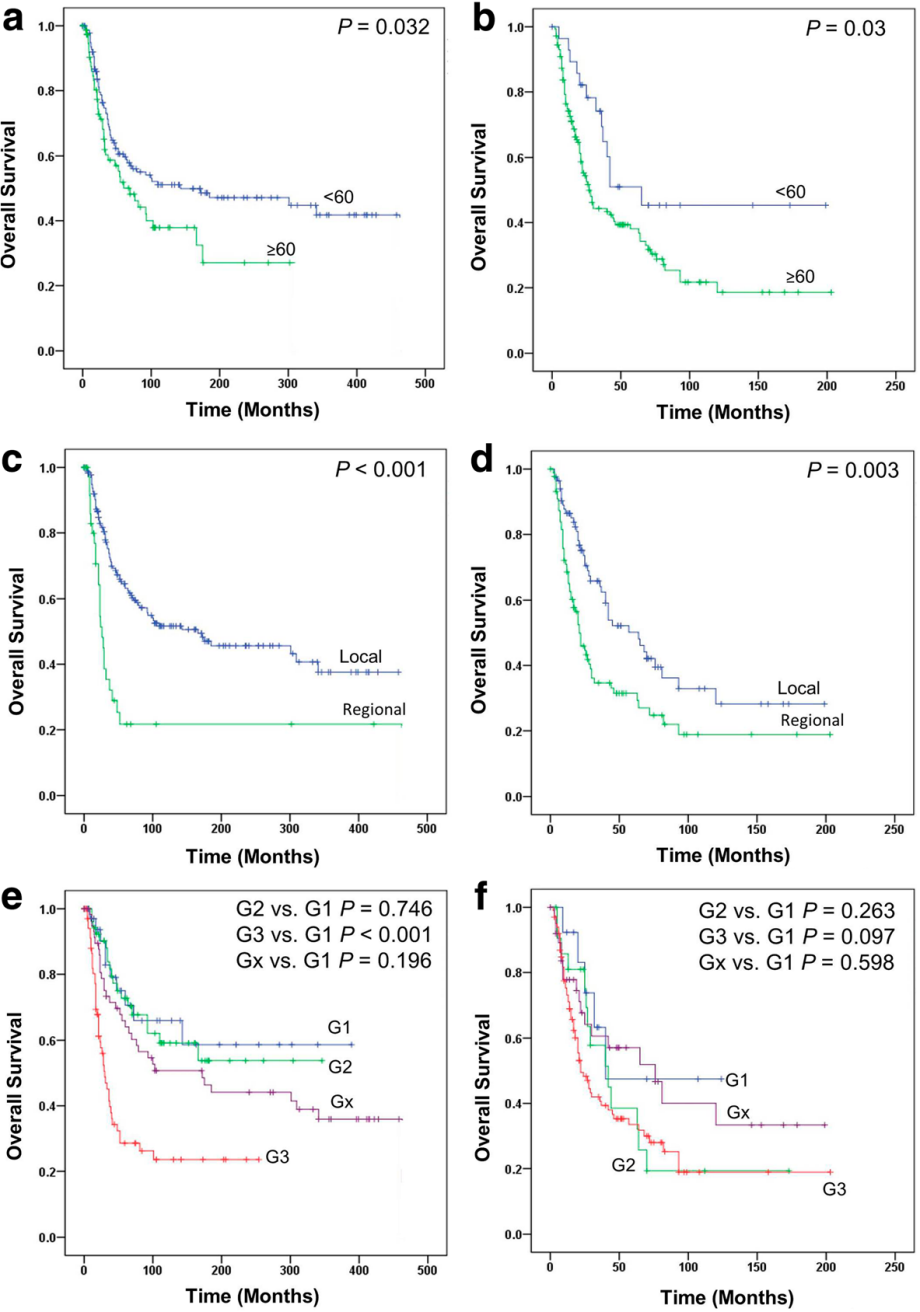
Kaplan-Meier survival *curves* in stratified analyses. **a** and **b**, *primary* and *secondary* breast angiosarcoma by age. **c** and **d**, *primary* and *secondary* breast angiosarcoma by tumor spread. **e** and **f**, *primary* and *secondary* breast angiosarcoma by tumor grade

We then examined the influence of those clinicopathologic characteristics as listed in Table 1 on overall survival outcomes of primary and secondary breast AS. We found that age and tumor spread were strong prognostic factors of poor OS in both primary and secondary breast AS. Advanced tumor grade (grade 3) was associated with significantly worse OS in primary AS and a tendency of worse OS in secondary AS (Table 2 and Fig. 1). In contrast, race, tumor anatomical site and number of primary tumors were not significantly associated with OS outcomes (Table 2).

**Table 2:** Association of clinicopathological characteristics and overall survival

		Primary			Secondary	
Parameters	HR	95% CI	*p*	HR	95% CI	*p*
Age (years)						
< 60	1			1		
≥ 60	1.54	1.04–2.28	0.032	1.91	1.07–3.42	0.03
Race						
Non-white	1			1		
White	1.47	0.85–2.53	0.169	1.33	0.62–2.87	0.464
Site						
Outer quandrant	1			1		
Inner Quandrant	0.82	0.43–1.58	0.549	1.51	0.68–3.34	0.307
Central	0.6	0.22–1.61	0.311	0.97	0.38–2.47	0.94
Overlap^a^	1.45	0.83–2.53	0.191	1.07	0.56–2.05	0.841
NOS^b^	1	0.56–1.77	0.988	0.75	0.43–1.32	0.315
Nipple					Not calculated	
Tumor spread						
Local	1			1		
Regional	2.66	1.69–4.19	<0.001	1.83	1.23–2.72	0.003
Tumor grade						
G1	1			1		
G2	1.14	0.53–2.44	0.746	1.79	0.65–4.98	0.263
G3	3.73	1.88–7.43	<0.001	2.16	0.87–5.34	0.097
Gx	1.6	0.79–3.26	0.196	1.31	0.48–3.52	0.598
Number of primaries^c^						
1 (2)	1			1		
≥ 2 (≥ 3)	1.04	0.64–1.70	0.866	1.3	0.88–1.93	0.191

^a^More than one quandrant but less than half breast size

^b^Entire breast: ¾ or more of breast involved with tumor, or multiple tumors in different subsites

^c^Comparisons were made in 1 versus ≥2 primary tumor groups in primary angiosarcoma and in 2 versus ≥3 primary tumor groups in secondary angiosarcoma

### Comparison of survival outcomes between primary and secondary breast AS

**Fig. 2:**
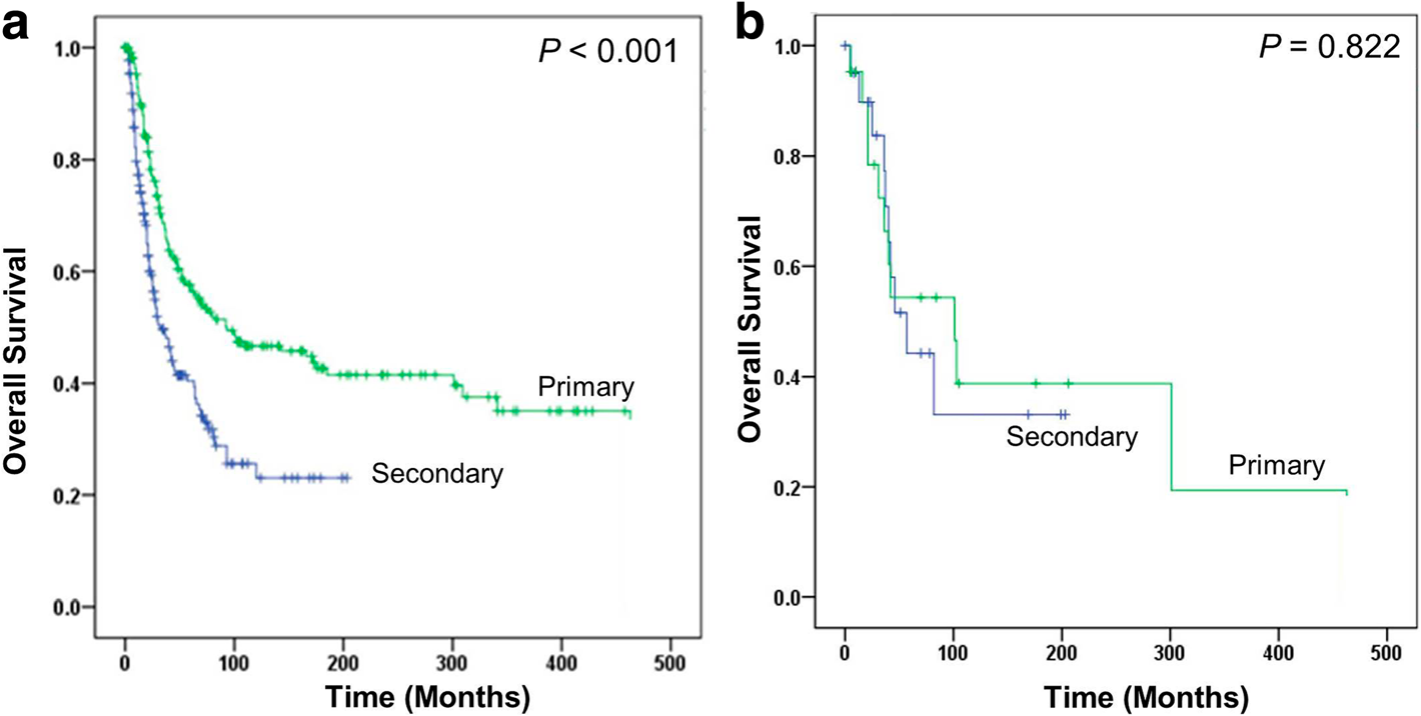
Kaplan-Meier *curves* to compare *primary* versus *secondary* breast angiosarcoma. **a** comparison in overall patient population. **b** comparison in matched group

As shown in Fig. 2a, patients with secondary breast AS had significantly worse survival outcome than patients with primary breast AS. The median OS were 93 and 32 months, and 5-year survival rates were 44.5 and 22.5%, respectively. A univariate Cox regression analysis showed a nearly 2 fold increased death risk of secondary breast AS, compared with primary breast AS (HR = 1.89, 95% CI, 1.43–2.50, *p* < 0.001). After adjustment with age, race, tumor grade, tumor stage, number of primary tumors, surgery and radiation history, patients with secondary breast AS seemed to have similar OS, compared with patients with primary breast AS (adjusted HR = 1.13, 95% CI, 0.74–1.72, *p* = 0.567).

To confirm the above result, we matched primary and secondary breast AS 1:1 by age, tumor stage, tumor grade and number of primary tumors. A total of 21 cases from secondary breast AS were matched with primary breast AS patients (Additional file 1: Table S1). Compared with primary breast AS, secondary breast AS showed similar death risk in matched group (HR = 1.11, 95% CI, 0.46–2.68, *p* = 0.822; Fig. 2b).

### Impact of treatment modality on survival outcomes

As with soft tissue sarcomas arising in other parts of the body, a multidisciplinary approach has been proposed to treat breast angiosarcoma. Since SEER does not collect chemotherapy information, we explored the impact of surgery and radiation therapy on survival outcomes. Among the 218 primary breast AS patients, 98.2% patients received surgery and 29.2% patients received radiation, most of which were given adjuvantly. Only 5 patients received radiation before surgery. Among the 173 secondary breast AS patients, 96.5% patients received surgery and 12.7% patients received radiation, all of which were given adjuvantly.

**Fig. 3:**
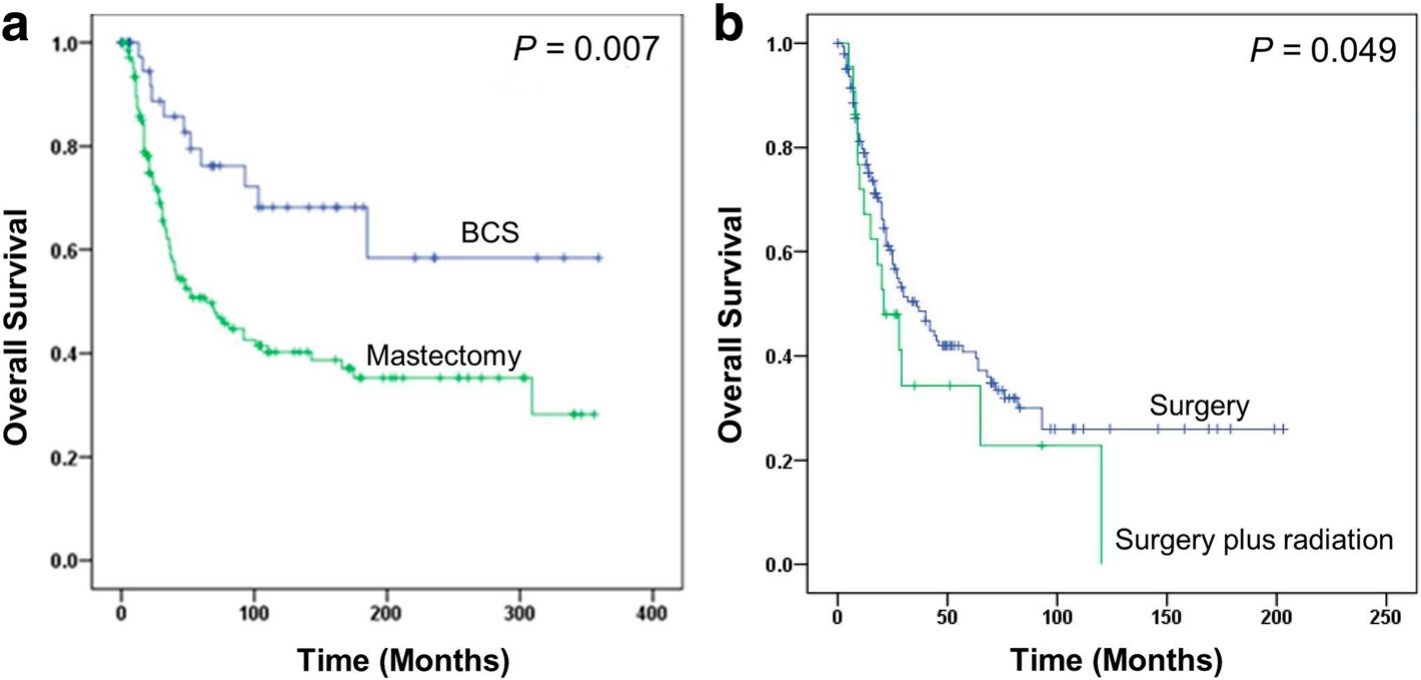
Kaplan-Meier curves by treatment modalities. **a** mastectomy versus breast conservative surgery in *primary* breast angiosarcoma. **b** radiation plus surgery versus surgery alone in *secondary* breast angiosarcoma

Since the majority of patients of both primary and secondary breast AS received surgery, we investigated if surgical modalities, breast conservative surgery (BCS) versus mastectomy, influenced survival outcomes. In primary breast AS patients, mastectomy was associated with significantly worse OS, compared with BCS (HR = 2.54, 95% CI, 1.35–4.79; adjHR = 2.47, 95% CI, 1.29–4.74) (Fig. 3a). However, in secondary breast AS patients, there was no significant difference in survival outcomes between BCS and mastectomy groups (Table 3). Compared with surgery alone, adding radiotherapy to surgery was associated a significantly increased death risk in secondary breast AS by multivariate analysis (adjHR =1.77, 95% CI, 1.01–3.12) (Fig. 3b), but not in primary breast AS.

**Table 3:** Associations of treatment modalities with overall survival in stratified analyses

	Crude HR	95% CI	*p*	Adjusted HR ^a^	95% CI	*p*
Surgery + radiation vs. surgery						
Primary	1.27	0.84–1.91	0.256	1.23	0.79–1.90	0.366
Secondary	1.38	0.80–2.40	0.25	1.77	1.01–3.12	0.049
Mastectomy vs. BCS						
Primay	2.54	1.35–4.79	0.004	2.47	1.29–4.74	0.007
Secondary	0.94	0.45–1.93	0.855	1.26	0.60–2.68	0.541

^a^ Adjusted by age, race, tumor stage, tumor grade and number of primaries

## Discussion

The rarity of breast AS precludes any prospective study and poses significant challenges in its diagnosis, treatment and research. The current comparative study of 218 primary breast AS and 173 secondary breast AS patients from SEER database represents the largest reported series to date. Our results showed that secondary breast AS usually presented at advanced age and advanced stage with aggressive tumor phenotype, which led to a “nominal” worse OS, compared with primary breast AS. Age, advanced stage and grade 3 of tumor seemed to be poor prognostic factors. Treatment modalities appeared to be associated with survival outcomes of primary and secondary breast AS.

Historically, breast AS was firstly reported in association with long-standing extremity edema (Stewart-Treves syndrome), and then was well known as a complication from radiation treatment. Published literature was limited by small sample-size, with a wide variation in description. The inconsistent information drawn from previous studies reflected a biased experience from small sample investigations and impairs the ability to draw firm clinical recommendations. Some clinicopathological features may influence survival outcomes of breast sarcoma, such as tumor grade, tumor size, tumor spread and margin status [3, 4]. In our study, age, advanced stage and grade 3 of tumor seemed to be prognostic factors of poor OS in both primary and secondary breast AS. Although grading of angiosarcoma is not recommended in current AJCC 7 for soft tissue sarcoma due to a presumed poor prognosis regardless of tumor grades, our results suggested that tumor differentiation could play a role in breast AS prognosis.

There is a common belief that breast AS has a bad prognosis, which is even worse in secondary breast AS. Our results showed median OS of 93 and 32 months, and 5-year survival rates of 44.5 and 22.5% for primary and secondary breast AS, which were consistent with previous reports [5–7]. Direct comparison of primary versus secondary breast AS showed an almost 2-fold increased death risk of secondary breast AS. The “nominal” increased death risk seems to be driven by older age at diagnosis and more aggressive tumor phenotype at presentation (e.g. advanced stage and higher tumor grade). If all the confounding factors were controlled, or if comparisons were made between matched groups, the survival outcomes seemed to be similar. This finding is important to correct the misconception that secondary breast AS is intrinsically worse than primary AS. In fact, primary and secondary breast AS seem to share similar underlying tumor biology and demonstrate comparable natural course.

There is no consensus of optimal treatment of breast AS and current recommendations for breast AS treatment are derived from small retrospective case reviews and extrapolated from non-breast soft tissue sarcoma studies [8]. Surgery, radiation and chemotherapy have all been used in breast AS, but there is a lot of debate regarding the surgical modalities and indication criteria of adjuvant therapy. In a review article of 100 secondary breast AS published in English literatures, the authors found over 73% patients developed tumor recurrences, the majority of which were within 1 year [9]. In another study of 35 secondary breast AS from North European cohort study, the authors found that 19 out of 23 patients developed local recurrence [10]. These results seemed to justify mastectomy in breast AS to prevent local recurrence. However, our analysis results showed that BCS was associated with better OS in primary breast AS and was at least non-inferior to mastectomy in secondary breast AS. Traumatic mastectomy may not be necessary in all breast AS, especially if there are no high risk features (e.g. large tumor size, deep location) and R0 resection can be achieved by BCS. This finding was supported by a recent study of surgical management of radiation-induced breast AS. In that study, the authors found that it was complete versus incomplete incision that made a difference in local recurrence or survival outcome, regardless of BCS or mastectomy [11].

In beast AS, several investigators routinely used adjuvant radiation in an attempt to improve local control. In a review article of 74 case reports consisting of 222 patients of secondary chest wall and breast angiosarcoma, the authors found that surgery with radiotherapy had a better 5-year local recurrence-free interval of 57% compared to 34% for surgery alone, which, however, did not translate into survival difference [12]. Confounding factors were not adjusted in this study because information was extracted from literatures. In our study, surgery plus radiation did not appear to improve survival outcome of unselected primary breast AS patients. Similar to the study aforementioned, there was no significant survival difference between surgery plus radiation and surgery alone groups in secondary breast AS by univariate analysis. However, adding radiation seemed to be associated with a worse survival outcome by multivariate analysis, which has not been reported before. Further examination of the data showed 20 out of 22 irradiated secondary AS patients received breast radiation in the past. It is not clear if this is a chance finding, or reflects a true harmful effect of re-irradiation in secondary breast AS patients over time, or is due to some unknown confounding factors which were not adjusted in our analysis. Indeed, patients receiving radiation treatment were more likely to have aggressive tumor biology, such as positive margin or lymphovascular invasion, and therefore might have a worse prognosis. However, patients who developed radiation-associated angiosarcoma might be intrinsically vulnerable to radiation damage, which could be the biological explanation for a harmful effect of re-irradiation in those patients. Nevertheless, the results from our and previous studies suggest that routine radiation in unselected breast AS should be cautious because there is at least no survival benefit. Attempt to control local recurrences by adjuvant radiation should be counterbalanced by the concern for later side effects.

Although this is the largest breast AS study to date, we recognize that our study has several inherent limitations. First, this is a retrospective analysis from SEER database, which is subject to the limitations of the number of variables collected. Some important variables, such as patient performance status and comorbidities were not collected and therefore could not be adjusted in our analyses. Second, SEER database does not report chemotherapy and tumor recurrence information. We were unable to evaluate impact of chemotherapy on survival outcomes and compare local recurrence rate by different treatment modalities. However, a study from MD Anderson showed reduced risk of local recurrence and a tendency of prolonged survival in patients receiving surgery plus chemotherapy [13]. Third, the SEER database does not differentiate cancer based on left or right position. Therefore, it is not possible to know if the secondary angiosarcomas developed ipsilateral to previously treated area. This is the limitation of the SEER dataset, which cannot be solved. Fourth, further analyses by cancer-specific survival were not performed because there are competing causes of death in patients with secondary breast AS who carry multiple malignancies. Hence, the documented death due to secondary breast AS in SEER database is not reliable to reflect its natural course. As a result, we included number of primary tumors as an adjusting factor in the analysis of overall survival outcomes.

## Conclusions

We demonstrated clinicopathological features of breast AS in the US population in a large population-based cohort study. Compared with primary breast AS, patients with secondary breast AS had a “nominal” increased death risk, which was driven by older age and more aggressive tumor phenotype at presentation. Both BCS and mastectomy are feasible in breast AS if R0 can be achieved. Routine radiation in unselected breast AS should be cautious because there is no survival benefit in primary AS and appeared to be associated with a worse OS in secondary AS. Further studies are needed in a prospective fashion.

## Additional file

Additional file 1: Table S1. This table provides clinicopathological characteristics of the matched primary and secondary angiosarcoma patients by age, race, tumor spread, tumor grade and number of primary tumors. (DOCX 14 kb)

## Abbreviations

AJCC: American Joint Committee on Cancer
AS: Angiosarcoma
BCS: Breast conservative surgery
CI: Confidence interval
HR: Hazard ratio
OS: Overall survival
RT: Radiotherapy
SEER: Surveillance, Epidemiology, and End Results Program
