# Supplementary material for: Prognosis and treatment of non-metastatic primary and secondary breast angiosarcoma: a comparative study
Source: BMC Cancer. 2017 Apr 27;17:295. doi: 10.1186/s12885-017-3292-7 (PMC5408408; doi:10.1186/s12885-017-3292-7)
Supplement: Additional file 1: Table S1. — This table provides clinicopathological characteristics of the matched primary and secondary angiosarcoma patients by age, race, tumor spread, tumor grade and number of primary tumors. (DOCX 14 kb) [file 12885_2017_3292_MOESM1_ESM.docx]

| **Supplementary Table** Patient characteristics (matched) | | |
| --- | --- | --- |
| **Parameters** | **Primary** | **Secondary** |
| Age (years) |  |  |
| <60 | 11 | 11 |
| ≥60 | 10 | 10 |
| Race |  |  |
| Non-white | 3 | 4 |
| White | 18 | 17 |
| Tumor spread |  |  |
| Local | 16 | 16 |
| Regional | 5 | 5 |
| Tumor grade |  |  |
| G1 | 0 | 0 |
| G2 | 4 | 4 |
| G3 | 12 | 12 |
| Gx | 5 | 5 |
| Number of primaries |  |  |
| 2 | 19 | 19 |
| 3 | 2 | 2 |
| Primary and secondary breast AS are matched 1:1 by age, tumor stage, tumor grade and number of primary tumors | | |
